# Beta-Papillomaviruses in Cutaneous and Mucosal Carcinogenesis: Oncogenic Mechanisms, Host Cofactors, and Clinical Significance

**DOI:** 10.3390/pathogens15101048

**Published:** 2026-10-02

**Authors:** Sayma Vizcarra-Ramos, Jesús García-Gómez, Wendoline Rojo-Contreras, Luis Felipe Jave-Suarez, Adriana Aguilar-Lemarroy

**Affiliations:** 1 Programa de Doctorado en Ciencias en Biomédicas, Centro Universitario de Ciencias de la Salud (CUCS), Universidad de Guadalajara, Guadalajara 44340, Jalisco, Mexico; sayma.vizcarra8588@alumnos.udg.mx (S.V.-R.); jesus.garcia9891@alumnos.udg.mx (J.G.-G.); 2 División de Inmunología, Centro de Investigación Biomédica de Occidente (CIBO), Instituto Mexicano del Seguro Social (IMSS), Guadalajara 44340, Jalisco, Mexico; luis.jave@academicos.udg.mx; 3 Hospital General de Zona No. 14, Instituto Mexicano del Seguro Social (IMSS), Guadalajara 44860, Jalisco, Mexico; elva.rojo@imss.gob.mx; 4 Unidad de Investigación Epidemiológica y en Servicios de Salud, Instituto Mexicano del Seguro Social (IMSS), Guadalajara 44340, Jalisco, Mexico

**Keywords:** beta-human papillomavirus, HPV, beta genus, oncogenic mechanisms, neoplasia, E6, E7, cancer

## Abstract

Human papillomaviruses of the beta genus (beta-HPV) are ubiquitous components of the cutaneous virome, persistently colonizing the skin of immunocompetent individuals from early infancy. Unlike their alpha counterparts, which drive mucosal carcinogenesis through viral integration and sustained oncoprotein expression, beta-HPVs employ transient, cofactor-dependent mechanisms that operate primarily at the initiation stages of keratinocyte carcinogenesis, a paradigm known as the hit-and-run model. Although their association with cutaneous squamous cell carcinoma is well supported, particularly in immunocompromised individuals, beta-HPV genotypes are being increasingly detected in patients with cervical, head and neck, anal, vulvar, and colorectal carcinomas. This raises the question of whether beta-HPVs play a broader oncogenic role. This review examines the clinical significance of beta-HPV across anatomical sites and evaluates existing and next-generation preventive strategies. Collectively, the evidence suggests that beta-HPVs may act as clinically relevant viral cofactors, although whether their detection at these sites is incidental or causal remains unclear.

## 1. Introduction

Historically, papillomavirus-driven oncogenesis was first established in animal models decades before an equivalent causal role was demonstrated for papillomavirus in humans. For instance, it has been demonstrated that bovine papillomavirus (BVP) is associated with progression to malignant carcinoma of the bladder and upper alimentary tract in cattle [1]. Furthermore, feline papillomavirus type 2 (FcaPV-2) has been associated with cutaneous squamous cell carcinoma (cSCC) in domestic cats [2]. These animal systems continue to provide a tractable framework for dissecting how cutaneous papillomavirus contributes to carcinogenesis in animals.

On the other hand, the human relevance of papillomaviruses is now well established: human papillomavirus (HPV) is considered one of the most prominent infectious agents attributable to cancer development worldwide, second only to *Helicobacter pylori,* and contributes to 31.1% of infection-induced cancers [3]. Alpha-HPV is the leading risk factor for cervical cancer, particularly when it is classified by the IARC as a Group 1 carcinogen (HPV16, 18, 31, 33, 35, 39, 45, 51, 52, 56, 58, and 59) [4]. In contrast, beta-HPV has emerged as a cofactor in the development of nonmelanoma skin cancer (NMSC), particularly cSCC, at sun-exposed sites [5,6].

Beta-HPV was initially believed to affect only immunocompromised individuals, such as those with epidermodysplasia verruciformis (EV) [7], and, consistent with this view, the IARC classified HPV5 and 8 as possible carcinogens (Group 2B) only within this clinical context [4]. However, given the increasing preclinical and epidemiological evidence indicating broader clinical relevance of beta-HPV across different immune states and anatomical sites [8,9], the IARC Monographs on the Identification of Carcinogenic Hazards to Humans have since called for its re-evaluation, with a focus on specific genotypes [10].

Current evidence indicates that the oncogenic mechanisms employed by beta-HPVs differ from those employed by alpha-HPVs. Rather than persisting in established tumors, beta-HPVs primarily exert their influence during the early stages of carcinogenesis, a mechanism described as the “hit-and-run” model, in which the virus initiates oncogenic changes but is not necessarily retained once the tumor is established [11,12].

Moreover, beta-HPV infections have been detected in a variety of carcinomas beyond the skin, including cervical, anal, vulvar, colorectal, and oral carcinomas [12,13,14,15,16]. This widespread detection raises the question of whether the presence of these viruses contributes to carcinogenesis at cutaneous and mucosal sites, a question that this review aims to address.

## 2. Methods

This article is a narrative review based on an electronic literature search conducted in the PubMed and Google Scholar databases. The search strategy combined MeSH descriptors and carcinoma-related terms using Boolean operators (AND/OR). The strategy used in PubMed was as follows: (“Betapapillomavirus” OR “Beta papillomavirus” OR “Beta-PV” OR “cutaneous HPV”) AND (“Neoplasms” OR “cancer” OR “carcinoma” OR “tumor” OR “malignancy”) AND (“prevalence” OR “incidence” OR “epidemiology” OR “seroprevalence” OR “detection” OR “association”).

This narrative review prioritized observational studies (cohort, case-control, or cross-sectional) that included human subjects of any age, sex, or immune status (immunocompromised or immunocompetent) and reported the detection of beta-HPV using validated molecular methods such as PCR, next-generation sequencing, or in situ hybridization in any type of cancer confirmed by histological methods or internationally accepted diagnostic criteria. In vitro and in vivo (animal models) studies were used to elucidate the underlying mechanism. Studies that did not distinguish between alpha and beta papillomaviruses were not considered. Because this is a narrative review based on the above search, it was designed to identify the representative literature rather than to provide a systematic or exhaustive synthesis; therefore, no formal record counts, PRISMA flow diagram, or risk-of-bias assessment are reported.

## 3. Beta-Human Papillomaviruses and Their Relationship with Nonmelanoma Skin Cancer (NMSC)

### 3.1. Overview of Human Papillomaviruses

Human papillomaviruses (HPVs) are double-stranded DNA viruses with a genomic structure of approximately 8000 bp. According to the HPV Reference Genome Center, 231 HPV genotypes have been genetically characterized to date. These genotypes are classified into five genera: alpha, beta, gamma, mu, and nu, distributed across the subfamily Firstpapillomavirinae of papillomaviruses (PVs). This classification is based on pairwise nucleotide sequence identity across the L1 open reading frame, and each genus is further subdivided into species, each exhibiting distinct features [13,14].

### 3.2. Beta-HPV as Part of the Skin Microbiome

The beta-human papillomavirus (beta-HPV) genus includes 54 recognized genotypes categorized into five species, beta-1 through beta-5 [14]. Beta-HPVs are commonly found in human skin and exhibit notable tropism for cutaneous tissues (Figure 1). Infection with these viruses typically occurs early in life through skin-to-skin contact and can persist throughout an individual’s lifespan [15]. In support of this, a longitudinal study evaluating forehead swab samples over a mean of 6.3 years revealed that 48% of healthy individuals harbored the same beta-HPV type at both time points, with beta-HPVs persisting more frequently than gamma-papillomaviruses (38% vs. 15%), and the most commonly detected persistent types were HPV20, HPV12, and HPV14D [16]. Hair follicles, particularly eyebrow hairs, have been described as important reservoirs for beta-HPV. In a longitudinal study of 23 healthy individuals over two years, beta-HPV DNA was detected in eyebrow hairs in 96% of participants, 74% of whom had persistent infection, with HPV23 being the most frequently detected type (74%), followed by HPV8 (39%) and HPV5 (26%) [17].

Specific genotypes, such as HPV5, 8, 23, and 47, have been detected in different parts of the body of healthy individuals, including the forehead, arms, and thighs [18,19]. Additionally, the Human Microbiome Project has identified other genotypes in the skin, including HPV75, 80, 151, 17, and 9 [20].

Beta-HPV possesses dual tropism, with certain genotypes detected not only in cutaneous sites but also in mucosal tissues typically associated with alpha-HPV [21]. For example, some authors have detected the presence of beta-HPV genotypes such as HPV5, 8, 38, 120, and 145 in other anatomical sites beyond the skin, including the oral cavity [22], anal mucosa [23], and external genital skin [24], of healthy individuals. In addition, the Human Microbiome Project has reported the presence of beta-HPV genotypes in the mouth (HPV17, 20, and 12) and vagina (HPV21, 38, 118, and 150) [20]. Notably, when beta-HPV infection was evaluated simultaneously across five anatomical sites within the same individual, the concordance of the same beta-HPV type was significantly greater across keratinized tissues (31.0%) than across mucosal sites (6.9%), and beta-3 genotypes showed a particular affinity for mucosal sites, suggesting that tissue tropism varies by species [25].

Furthermore, Winer et al. reported a prevalence of 20.6% of beta-HPV in oral samples from healthy women, with the most prevalent genotypes being HPV23, 38, 24, 111, and 124 [26]. In another study from the same research group, the prevalence of beta-HPV in fingernails was reported to be higher than that in oral samples (61.1%). The most common genotypes were HPV23, 38, 5, 107, and 120 [27].

An interesting point of these studies is that they associate the presence of beta-HPV in oral samples with fingernail samples from the same individual, reporting a strong association between both, which supports the hypothesis that fingernails are a source of transmission and autoinoculation of these viruses in the oral cavity [26,27].

**Figure 1:**
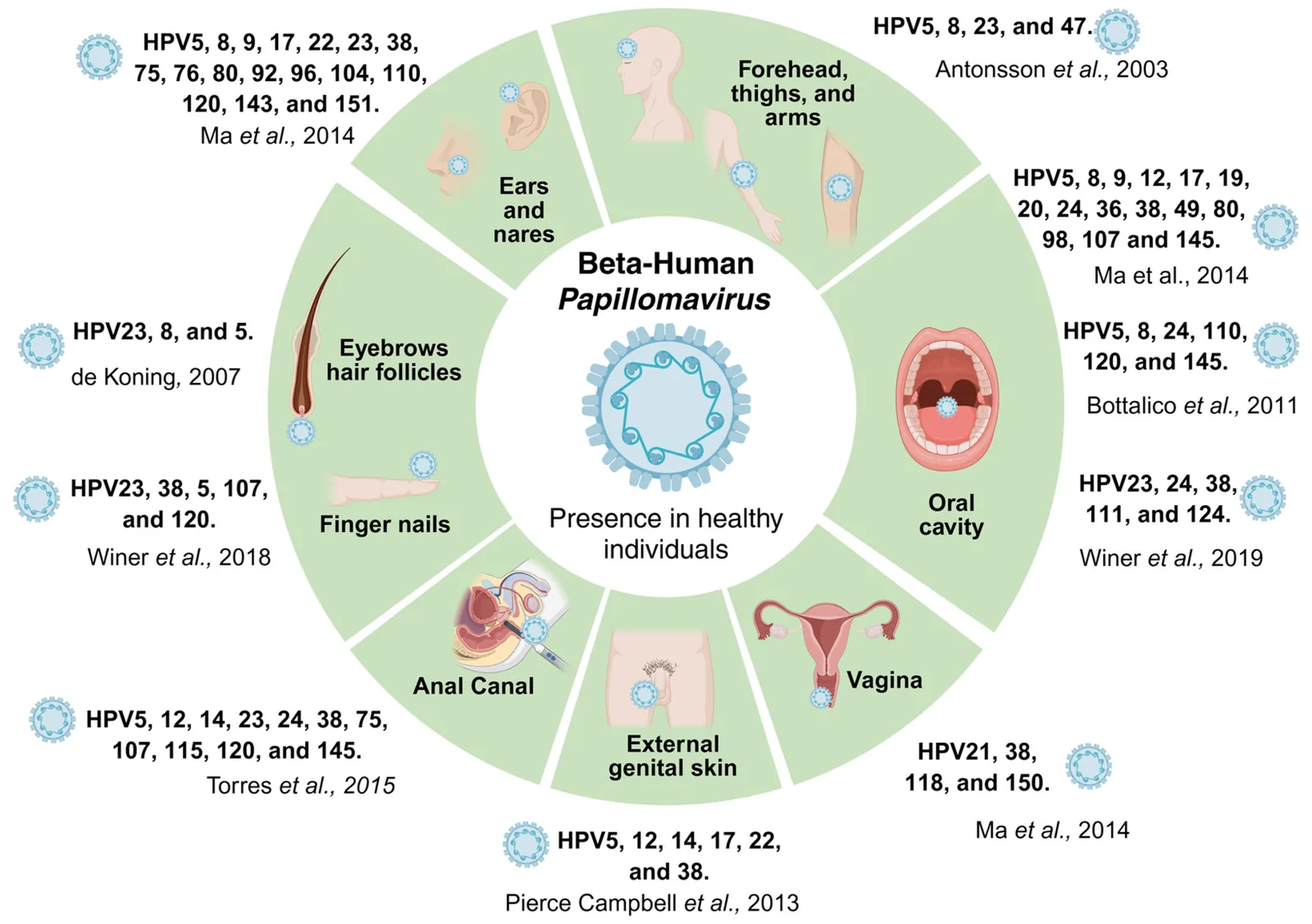
Beta-human papillomavirus (beta-HPV) is a ubiquitous commensal. The detection of beta-HPV infection across different anatomical sites in healthy individuals was performed according to the methods in the referenced publications [17,19,20,22,23,24,26,27]. Created in BioRender. Vizcarra Ramos, S. (2026) https://BioRender.com/nr2h6nj (accessed on 22 July 2026).

### 3.3. The Association of Beta-HPV with NMSC

While beta-human papillomavirus is typically harmless in healthy individuals, it can act as a cofactor in the development of NMSC, particularly in immunocompromised individuals. This association was first identified in patients with epidermodysplasia verruciformis (EV), and the first genotypes classified in the genus, HPV5 and 8 (Beta-1 species), were isolated from the skin lesions of individuals diagnosed with this disease [7].

A recent systematic review reported the prevalence of beta-HPV infection in patients with various skin conditions. For instance, in the case of actinic keratosis, cSCC, the prevalence was 58.49%. In cSCC, it was 55.98%, which is greater than the prevalence of infection with the gamma and alpha genera [28].

Similarly, organ transplant recipients are particularly susceptible to developing beta-HPV-associated malignant cutaneous lesions, especially in sun-exposed areas [29]. A large multicenter prospective cohort study confirmed this association, demonstrating that organ transplant recipients harboring five or more beta-HPV types in eyebrow hair had a 1.7-fold increased risk of developing cSCC, with a high viral load conferring a similar risk (hazard ratio, HR 1.8; 95% CI 1.2–2.8) [30]. These findings highlight the combined impact of immunosuppression and ultraviolet radiation exposure in facilitating beta-HPV-related carcinogenesis. Nonetheless, the increased risk of beta-HPV-related carcinogenesis is not limited to immunocompromised individuals. Immunocompetent individuals harboring beta-HPV infections face a 42% increased risk of developing cSCC, with HPV5, 8, 15, 17, 20, 24, 36, and 38 identified as the highest risk genotypes [9]. Prospective evidence from the VIRUSCAN cohort further supported this association, demonstrating that baseline beta-HPV detection in forearm skin swabs significantly predicted subsequent cSCC development (HR 4.32, 95% CI 1.00–18.66) and that beta-HPV-positive cSCC tumors occurred more frequently in areas of highly sun-damaged skin, reinforcing the cooperative role of viral infection and ultraviolet radiation in cutaneous carcinogenesis [31].

While immunosuppression plays a critical role in increasing the risk of beta-HPV-associated carcinogenesis, it is not the only contributing factor. Additional mechanisms are involved in the development of beta-HPV-related cancers. However, the precise molecular and cellular pathways underlying beta-HPV-induced carcinogenesis remain incompletely understood and are the focus of ongoing research.

## 4. Contrasting the Oncogenic Mechanisms of Alpha- and Beta-HPV

### 4.1. HPV Genome: Alpha vs. Beta

The genome structure of all alpha-HPVs consists of three major regions. The first is the long control region (LCR), which plays a regulatory role in viral replication and transcription. The second is the early region, encoding proteins E1, E2, E3, E4, E5, E6, and E7; these proteins are involved in viral replication, cell transformation, and modulation of the host’s cellular environment. The third region is the late region, encoding the L1 and L2 structural proteins essential for the formation of the viral capsid [11].

In contrast to alpha-HPV, beta-HPV lacks an open reading frame (ORF) for the E5 oncoprotein, which enhances the immortalization effects of E6 and E7 and promotes metastasis in alpha-HPVs. This absence suggests that beta-HPV E6 and E7 may have functionally diverged to compensate for the lack of E5 [32]. Another key distinction lies in the LCR region, which is shorter in beta-HPVs (~478 bp) than in alpha-HPVs (~800 bp) [11].

### 4.2. Infection Patterns of Alpha- and Beta-HPV

Alpha-HPV is commonly detected in the anogenital region of men and women, with the cervix being the most frequent site of infection. The host immune system clears most cervical alpha-HPV infections within one to two years. However, if unresolved, they become persistent, leading to the integration of the *E6* and *E7* genes into the host genome and initiating carcinogenesis [32].

In contrast, beta-HPV infections are typically shorter, with a median persistence of 8.6 months in eyebrow hair and 11.3 months in skin swabs, and viral integration has not been observed [33]. This suggests that its role in carcinogenesis is transitory and, unlike alpha-HPVs, does not require constant expression of the oncoproteins E6 and E7 [6,11].

### 4.3. Mechanisms of E6-Driven Oncogenesis in Alpha- and Beta-HPV

Alpha- and beta-HPV strains exhibit crucial differences not only in their genome structure but also in their oncogenic mechanisms (Figure 2). The function of the E6 protein in alpha-HPV, particularly among high-risk (HR) genotypes, is well characterized. This protein induces the degradation of p53, a tumor suppressor that regulates the cell cycle, DNA repair, and apoptosis. The E6 protein binds to E6-associated protein (E6AP), forming a complex that subsequently binds to p53. By utilizing the E3 ubiquitin ligase function of E6AP, p53 is ubiquitinated and degraded by the proteasome. The loss of p53 is a pivotal event in virus-induced cellular transformation [6].

In contrast, the beta-HPV E6 protein typically inactivates p53 through E6AP-independent mechanisms. For example, upon UV-induced DNA damage, HPV23 and HPV38 E6 (both beta-2 species) bind to homeodomain-interacting protein kinase 2 (HIPK2), a kinase that phosphorylates p53 at Ser46, triggering pro-apoptotic transcription; by enforcing dissociation of the HIPK2/p53 complex, E6 prevents this phosphorylation event and inhibits UV damage-induced apoptosis [34]. Alternatively, E6 of HPV17, 38, and 92 binds directly to p53, stabilizing it and altering its transcriptional activity [35]. Additionally, HPV38 E6 increases the expression of the N-truncated isoform of p73 (ΔNp73), a dominant-negative antagonist of p53 [36]. Notable exceptions exist within beta-3 species, such as HPV49, 75, and 76, which can form the E6AP complex and degrade p53 via the proteasome, mimicking alpha-HPV mechanisms [37].

Beta-HPVs also drive oncogenesis in concert with UV-induced DNA damage by interfering with host cell mechanisms involved in apoptosis, DNA damage repair (DDR), cellular proliferation, and immortalization [38].

Following UV damage, HPV genotypes such as HPV5 and 8 (beta-1 species), as well as the beta-2 genotype 38, promote genomic instability through distinct mechanisms involving the histone acetyltransferase p300. HPV5 and HPV8 E6 block the phosphorylation of p300 by AKT, resulting in p300 destabilization; the consequent reduction in p300 activity impairs ATM/ATR signaling, downregulates BRCA1/2 expression [39], compromises double-strand break repair and UV-damage responses, and increases genomic instability [40]. In contrast, HPV38 binds to p300 without destabilizing it, instead preventing p53 acetylation and inhibiting its transcriptional activity, thereby impairing p53-mediated responses to UV-induced DNA damage [41].

Moreover, these viruses facilitate the survival of damaged cells through the evasion of apoptosis. Following UV-induced DNA damage, E6 of HPV5, 8, 20, 22, 76, 92, and 96 triggers the proteasomal degradation of Bak, a pro-apoptotic BCL2 protein essential for initiating cell death under UV-induced cellular stress. The degradation of Bak facilitates the accumulation of DNA mutations and the survival of damaged cells [42].

Beyond disrupting DNA integrity and apoptosis, beta-HPVs also alter cell differentiation pathways, notably through the dysregulation of NOTCH signaling, a pathway closely associated with cSCC development [6]. For instance, E6 proteins from multiple beta-HPV types, including HPV8, 17, 38, 76, and 92, bind to the C-terminal acidic domain of Mastermind-Like Transcriptional coactivator 1 (MAML1) via an LDDLL motif, repressing MAML1-mediated Notch transcriptional activation and the expression of endogenous Notch target genes such as *HES1*, *HEY1* and *NRARP*. This inhibition of Notch1-regulated gene expression alters keratinocyte differentiation, maintaining cells in a proliferative, basal state and promoting tumorigenesis [43,44]. Another signaling pathway associated with cellular differentiation is Hippo signaling; HPV8 E6 activates the transcriptional enhanced associated domain (TEAD), subverting the signaling pathway. Additionally, E6 interferes with Wnt signaling by binding to the β-catenin-associated transcriptional coactivator B-cell CLL/lymphoma 9-like (BCL9L) [45].

Finally, emerging evidence indicates that infection with HPV38, 49, 75, and 76 immortalizes human foreskin keratinocytes, mainly through the increased transcription of human telomerase reverse transcriptase (hTERT) mediated by E6 (Table 1) [37,46,47].

### 4.4. Mechanisms of E7-Driven Oncogenesis in Alpha- and Beta-HPV

The E7 oncoprotein typically targets pRb and other proteins of the retinoblastoma family. In HR alpha-HPVs, E7 mediates the proteasomal degradation of pRb by recruiting the cullin-2 ubiquitin ligase complex [32]. pRb is a key regulator of the cell cycle that controls the G1 to S phase transition. In its active, unphosphorylated state during early G1, pRb binds and inhibits E2F transcription factors, preventing the expression of S-phase genes. As the cycle progresses, pRb is progressively phosphorylated, releasing E2F, which in turn activates the transcription of genes required for DNA synthesis. By degrading pRb, the E7 oncoprotein liberates E2F, leading to the upregulation of cell cycle promoters such as CDK2 and cyclins A and E, which leads to uncontrolled cell cycle progression [52] (Figure 2).

In contrast to their alpha-HPV counterparts, beta-HPV E7 proteins have not been shown to induce the proteasomal degradation of pRb. However, E7 proteins from multiple beta-HPV types, including HPV38 and beta-3 species, such as HPV49, 75 and 76, can inhibit pRb function through hyperphosphorylation, which is a less permanent form of inhibition than HR alpha-HPV-mediated proteasomal degradation [37,50]. Furthermore, the HPV38 E7 oncoprotein, like E6, induces the accumulation of ΔNp73, thereby inhibiting p53-mediated apoptosis. HPV38 E7 also promotes the nuclear translocation of IκB kinase beta (IKKβ), which phosphorylates and stabilizes ΔNp73α. Nuclear IKKβ and ΔNp73α subsequently recruit the epigenetic enzymes DNA methyltransferase 1 (DNMT1) and enhancer of zeste homolog (EZH2), forming a tetrameric inhibitory complex (IKKβ/ΔNp73α/DNMT1/EZH2) that silences a subset of p53-regulated promoters involved in DNA repair and apoptosis, such as PIG3, through histone H3 lysine 27 trimethylation and DNA methylation (Table 1) [51].

### 4.5. The “Hit-and-Run” Mechanism

The oncogenic mechanism of beta-HPV E6 and E7 described above converges on a central concept: these viruses appear to act exclusively during the early stages of carcinogenesis. In this mode, known as the “hit-and-run” mechanism, beta-HPVs increase the mutagenic capacity of cells to respond to UV radiation, promoting the survival and proliferation of DNA-damaged keratinocytes. Once oncogenic driver mutations are established, the virus is no longer necessary for the maintenance of the malignant phenotype and may be progressively lost; consequently, not all neoplastic cells contain a copy of viral DNA, and viral integration into the cellular genome is not observed [47].

There is substantial evidence that supports this paradigm. First, the viral load of beta-HPVs, such as HPV5, 8, 15, 20, 24, and 36, is significantly greater in precancerous actinic keratoses than in established cSCC and basal cell carcinomas, with viral loads falling below the detection limit in 32 of 79 HPV-positive biopsies, indicating that not all cells within a lesion harbor at least one viral copy [53]. Additionally, although beta-HPV DNA was detected in cSCC samples, transcriptome sequencing failed to identify any viral mRNA expression in these tumors, demonstrating that viral protein expression is dispensable for the maintenance of the malignant phenotype [54]. Experimental models further corroborate this mechanism. In a natural animal model (*Mastomys coucha* infected with MnPV), UVB irradiation and papillomavirus infection cooperatively promoted cSCC in 56% of infected animals versus 5% of uninfected controls; critically, viral DNA and transcription decreased as tumors progressed toward a more malignant phenotype, yet these virus-negative tumors remained seropositive for MnPV, confirming prior infection and demonstrating that viral presence becomes dispensable once malignant progression is established [55]. Consistent with these findings, studies in transgenic mouse models have confirmed that HPV38 E6 and E7 synergize with UV-induced DNA mutations to initiate skin lesions, but viral oncoprotein expression is not required to maintain the malignant phenotype [12]. Taken together, these findings support a model in which beta-HPVs contribute to skin carcinogenesis through a hit-and-run mechanism, acting at early stages but becoming dispensable once oncogenic driver mutations are established [56].

### 4.6. Immune Surveillance Rather than Viral Hit-and-Run

An alternative explanation for the “hit-and-run” mechanism is that the absence of detectable beta-HPV in tumors reflects active immune-mediated elimination of virus-infected cells during tumor development. Recent studies in mouse models and human tissue have demonstrated that beta-HPV colonization induces a robust CD8+ T-cell immune response [57,58].

Strickley et al. proposed that active infection with beta-HPV activates the immune system, eliminating positive beta-HPV cells through tumor development and explaining why the viral load decreases in established tumors [58].

Additionally, Son et al. demonstrated in mouse models and human tissue that beta-HPV colonization induces a robust CD8+ T-cell immune response. In immunocompetent hosts, virus-specific CD8+ T cells (particularly CD103+ tissue-resident memory T cells) recognize and eliminate keratinocytes that harbor beta-HPV and have acquired TP53 mutations. Because these p53-mutant keratinocytes exhibit increased viral activity and antigen presentation, they become targets for immune clearance [57].

However, Wallace and Akgül, emphasize that under immunosuppressive UV doses, the same virus can promote rather than prevent tumorigenesis and that cutaneous HPVs are heterogeneous (for example, unlike MMuPVI, HPV5 and HPV8 can degrade p300 and impair DNA repair). Variations in immune status, viral load, and beta-HPV diversity must be better understood before therapies targeting beta-HPV immunity can be translated into clinical practice [59].

## 5. Beta-HPV Oncogenesis: Collaboration with Environmental and Immune-Related Host Factors

The evidence presented earlier in this review indicates that beta-HPV and UV exposure act together in the development of cSCC, suggesting that these viruses require the assistance of cofactors. A connection exists between the persistence of beta-HPV infection and genetic disorders, host immune responses, and other environmental cofactors, which will be addressed in the following sections and are summarized in Table 2.

### 5.1. Relationships Between Genetic Alterations and Immunosuppression

The association between beta-HPV infection and the development of NMSC was first discovered in patients with EV, a rare recessive autosomal genetic disease associated with mutations in the genes *EVER1* (*TMC6*) and *EVER2* (*TMC8*). These genes interfere with the epithelial antiviral response, enabling the persistence of HPV5 and 8 infections [7,11].

EVER proteins are presumably involved in the regulation of cellular zinc balance in keratinocytes and T and B lymphocytes; dysfunctions in these proteins lead to an impairment in the immune response to beta-HPV infection [60]. Specifically, it has been demonstrated that EVER2 blocks the LCR of HPV5, thereby stopping its replication process, as EVER2-deficient cells activate this region through a c-Jun N-terminal kinase (JNK)-dependent pathway. Moreover, the EVER2 protein regulates TRAIL-dependent apoptosis, which facilitates the elimination of HPV-infected cells through the production of TNF-α and TRAIL; therefore, EV patients have difficulty eliminating infected cells because of defects in this protein [61,75].

In addition, patients with EV also exhibit mutations in the *CIB1* gene, which encodes calcium- and integrin-binding protein 1. The EVER1/EVER2 complex partners with CIB1 to suppress beta-HPV replication, whereas other HPV genera have evolved proteins that interact with and overcome CIB1-mediated restriction: alpha-HPVs through their E5 protein (as demonstrated for HPV16) and gamma-HPVs through their E8 protein (as demonstrated for HPV4); beta-HPVs lack both E5 and E8 and therefore cannot overcome this restriction. Consequently, a deficiency in the CIB1-EVER1-EVER2 complex compromises keratinocyte-intrinsic defense against beta-HPV, making EV patients selectively susceptible to infection with these types [62].

On the other hand, EVER mutations are not the only genetic modifications associated with beta-HPV infection. Antonsson et al. evaluated the associations between specific SNPs and beta-HPV seropositivity, linking, for instance, the rs9264942-T allele with HPV 9, 15, and 17. This polymorphism is involved in the regulation of HLA-C, which is essential for the activation of immune effector cells, such as cytotoxic T lymphocytes, which eliminate virus-infected cells [63].

### 5.2. Immune Evasion Mechanisms of Beta-HPVs

The persistence of beta-HPV infection is closely related to an impairment in immune responses. The innate immune system orchestrates the first line of defense against HPV, beginning with the recognition of the virus by Toll-like Receptor 9 (TLR9), a sensor of double-stranded DNA within the keratinocyte, leading to the activation of different signaling pathways that eliminate infected cells [64].

Beta-HPV, especially HPV38, evades the innate immune system by downregulating the expression of *TLR9*. The oncoproteins E6 and E7 induce the accumulation of ΔNp73α, which acts as a negative regulator of *TLR9* transcription [64]. Moreover, this mechanism is related to UV radiation. The cells affected by UV radiation activate TLR9 as part of the cellular stress response, recruiting p53 and c-Jun. Nonetheless, HPV38 prevents this recruitment, preventing TLR9 activation [65].

Additionally, HPV38 evades innate immunity by suppressing the expression of IL-18, a proinflammatory cytokine produced downstream of inflammasome activation in response to UV-induced cellular stress. Under normal conditions, UV exposure activates p53, which binds the IL-18 promoter and drives its transcription; mature IL-18 is then generated following inflammasome-mediated cleavage of pro-IL-18, amplifying the immune response to eliminate UV-damaged cells. However, HPV38 E6 and E7 prevent p53 recruitment to the IL-18 promoter and instead recruit an inhibitory complex (ΔNp73α/PKR/DNMT1) that silences IL-18 transcription, thereby dampening this protective immune response [66].

Another antiviral mechanism of the innate immune system involves the production of type I and II interferons. The signaling pathway of these cytokines is also impaired during HPV38 infection, as the E6 and E7 oncoproteins of the virus decrease the expression of STAT1 (an important transcription factor for IFN-I and II production). On the other hand, low expression of STAT1 results in lower levels of downstream effectors, such as IRF1 and TAP. The latter leads to a reduction in the expression of MHC I, which affects antigen presentation in the adaptive immune response [67].

Additionally, it has been demonstrated that HPV5 and 8 suppress the induction of IFNβ (a type I IFN). To induce the production of IFNβ, the transcription factor IRF3 needs to be phosphorylated and translocated to the nucleus. In HR-HPV, such as HPV16, E6 can directly bind to IRF3 and inhibit the production of interferons. However, the beta-HPV mechanism indirectly inhibits this production; HPV5 and HPV8 E6 interact with a coactivator protein of IRF3, CREB binding protein (CBP) [76]. Moreover, HPV8 E6 attenuates cyclic GMP-AMP synthase stimulator of interferon genes (cGAS-STING) signaling by reducing STING phosphorylation in response to cytosolic dsDNA, broadly downregulating innate immune gene expression and ISG production [68].

Finally, beta-HPV can evade host immune surveillance by inhibiting the migration of Langerhans cells (LCs), a type of antigen-presenting cell that is restricted to the epithelium. This phenomenon, along with a reduction in the expression of CCL20 (a chemoattractant of Langerhans cells), has been observed in patients with EV, where HPV8 plays an important role [69]. In this case, oncoprotein E7 of HPV8 interacts with the transcription factor “CCAAT/enhancer-binding protein β (C/EBPβ)”, which in human keratinocytes regulates the transcription of CCL20, thus reducing the expression of the chemokine and the migration of the LC [69].

### 5.3. Chronic Inflammation in Beta-HPV Infection

The viral life cycle of beta-HPV leads to an inflammatory microenvironment at the infection site, resulting from the immune system’s attempt to clear the virus. Nonetheless, persistent infection can result in a chronic inflammatory response, potentially contributing to the promotion of skin carcinogenesis, particularly in immunocompromised individuals, such as those with EV [75].

Evidence suggests that beta-HPV infection can increase cytokine production; for example, HPV5 upregulates the expression of IL-6, IL-8, ICAM-1, and MCP-1 (CCL2), thereby facilitating an antiviral inflammatory response [70]. However, in immunocompromised settings such as EV, specific cytokines may promote tumorigenesis. For instance, Vella et al. reported that the expression of the chemokine CCL2, which is mediated by the E6 oncoprotein of HPV8 through the C/EBPα/*miR-203*/p63 pathway, is strongly upregulated in the lesional epidermis of EV patients, leading to CCR2-dependent monocyte recruitment and macrophage infiltration in both the stroma and epidermis of EV lesions [71]. Importantly, this monocyte recruitment might not be merely a bystander effect but a causal driver of carcinogenesis: HPV8 E6 transgenic mice crossed with Ccr2^−/−^ mice (which lack circulating Ly6Chi monocytes) were completely resistant to UV-induced cSCC and showed no signs of epidermal hyperplasia or dysplasia, whereas HPV8-E6 mice with intact monocyte recruitment developed tumors [77].

Additionally, Podgórska et al. reported that the E2 protein of HPV8 can increase the expression of the proteins S100A8 and S100A9 by interacting with C/EBPβ. S100A8 and S100A9 form a complex known as calprotectin, which is related to chronic and tumor-promoting inflammation in skin carcinogenesis. This complex recruits neutrophil granulocytes to the site of infection (cells frequently present in the lesions of EV patients), thus contributing to a protumorigenic environment in these patients [72].

### 5.4. Environmental Cofactors in Beta-HPV-Mediated Carcinogenesis

To date, the only cofactor besides UV with direct experimental evidence of a role in potentiating beta-HPV carcinogenesis is tobacco. A transgenic mouse model expressing HPV49 E6 and E7 in the basal layer of mucosal epithelia of the digestive tract demonstrated high susceptibility to upper digestive tract carcinogenesis upon exposure to 4-nitroquinoline 1-oxide (4NQO), a surrogate for tobacco-associated carcinogens; in contrast, compared with control mice, HPV38 E6/E7 transgenic mice showed little susceptibility to 4NQO but remained highly susceptible to UV-induced skin carcinogenesis [74]. A subsequent study confirmed that cancer development in HPV49 E6/E7 transgenic mice exposed to 4NQO correlated with mutational signatures characteristic of tobacco exposure, suggesting that HPV49, along with tobacco, may influence oropharyngeal and esophageal carcinoenic processes [73].

## 6. Clinical Significance: Reported Detection of Beta-HPV Infections in Other Cancers

### 6.1. Cervical Cancer

Alpha-HPV infection, specifically with HR genotypes, is associated with 90% of cervical and anal carcinomas, 40–60% of penile and vaginal carcinomas, and up to 60% of oropharyngeal squamous cell cancers [78]. Nonetheless, the presence of beta-HPV infections in the anogenital region of healthy men and women has raised questions regarding a possible association between these infections and cancer development at these anatomical sites (Figure 3).

Multiple research groups worldwide have reported the presence of beta-HPV infections in the cervix. One of the first studies was conducted in a Brazilian population (Ludwig-McGill cohort), in which beta-HPV infection (27.3%) was more frequent than alpha-HPV infection (21.6%) among cervical samples from 505 women. The most prevalent beta-HPV genotypes included HPV21, 22, and 38. However, these infections tend to be transient, as only 1.98% of infections persisted after twelve months, and no association with cervical cytological abnormalities was found [79]. Moreover, Malagon et al. reported in the same cohort additional beta-HPV genotypes among the most prevalent infections, such as HPV5 and 8. They also reported that coinfections with other beta-HPVs occurred 2.24 times more often than expected, while coinfections with alpha-HPV genotypes occurred 0.64 times more frequently. Despite these findings, evidence does not currently support a correlation between the presence of beta-HPV infection or coinfection and the development of cervical abnormalities [80].

Interestingly, a study conducted in 138 HIV-positive women in Italy revealed that the prevalence of beta-HPV infection in cervical samples is 30.3%. The most frequent infections were with beta-1 species (HPV5, 12, and 47); however, they also reported infections with beta-2 species (HPV38, 15, and 111) and beta-3 species (HPV76). Like Malagon et al., the authors of this study reported that there was no statistically significant association between beta-HPV infections and neoplastic precancerous lesions such as LSIL and HSIL [81].

Using next-generation sequencing (NGS) with universal oligonucleotides (PGMY and FAP), our research group detected alpha, beta, and gamma genotypes in cervical cancer samples from Mexican women, identifying the beta-HPV types HPV38b (16.7%), HPV107 (7.8%), and HPV122 (3.3%) as the most prevalent [82]. Subsequent functional characterization revealed that E6/E7 from HPV122 exhibited proliferative, migratory, and invasive capacities comparable to those of HPV16 and HPV18 in primary human fibroblasts, whereas those of HPV38b and HPV107 extended the cellular lifespan and enhanced migration; all three genotypes induced ΔNp73 accumulation and downregulated the p53 target genes *PTCHD4* and *CDKN1A* [92]. More recently, E6/E7 from HPV80 was also shown to extend fibroblast lifespan and enhance proliferation, with transcriptomic analysis revealing enrichment in *p53*, *TNF*, and chemokine signaling pathways shared with HPV16 [93]. While these findings provide the first functional evidence that beta-2 HPVs detected in cervical cancer possess oncogenic potential in vitro, the current evidence remains insufficient to establish a causal relationship, and independent validation in larger cohorts and appropriate tissue models is needed. Additionally, Dom-Chima et al. reported the presence of beta-HPV infections in cervical cancer samples in the Nigerian population, with a prevalence of 7% for HPV20, which ranked among the five most prevalent types. Additional beta-HPV genotypes, including HPV14, 21, 118, 152, and 196, were also identified. This raises the question of whether the presence of beta-HPV genotypes in patients with cervical cancer contributes to carcinogenesis or is merely incidental [83].

### 6.2. Head and Neck Cancer (HNC)

In 2014, Odar et al. evaluated the presence of beta-HPV infections in 30 head and neck verrucous carcinomas and reported a prevalence of 16.7%, specifically for HPV19, 24, and 36. The authors suggest that the presence of beta-HPV could be incidental colonization; however, they also mention that it may have a biological function in this cancer [90]. On the other hand, Agalliu et al. conducted a study using samples of head and neck squamous cell carcinoma (HNSCC, which includes the oropharynx, oral cavity, and larynx). They reported that beta-HPV infection was present in 58.8% of controls and in 62.1% of cancer patients, particularly those with the genotypes HPV5, 17, and 38. The authors suggest a possible role of different HPV genotypes (not only beta but also alpha and gamma-HPV) in HNSCC etiology; however, further studies are needed to confirm the hypothesized role [89].

Moreover, Sabol et al. reported the presence of beta-HPV in oral lesions, oral cancer, and oropharyngeal tumors, as well as in healthy individuals. The prevalence in oral lesions (67.7%) was greater than that in healthy individuals (56.7%) and oral cancer (55.2%), whereas oropharyngeal tumors (45.7%) were less common. The most frequent HPV genotypes found were HPV5, 38, and 122; HPV5 and HPV122 were significantly associated with HNC, suggesting a possible etiological role that must be confirmed in future research [91]. In contrast, a large multicenter case-control study in Iran (IROPICAN) revealed no significant association between beta-HPV infection and HNSCC risk (adjusted odds ratio [aOR] 1.18, 95% CI 0.9–1.6), despite the detection of beta-HPV in 43.8% of patients and 38.6% of controls, with HPV23 (8.9%), HPV38 (7.9%), HPV24 (6.1%), HPV5 (6.1%), and HPV49 (6.0%) as the most prevalent types in patients across the oral cavity, larynx, and pharynx subsites [87].

In addition, beta-HPV infection has been found in rare head and neck cancers, such as middle ear squamous cell carcinoma (MESCC). The authors of this study reported that 66.7% of the patients had an HPV infection, and the prevalence of beta-HPV infection was greater (63.6%) than that of alpha-HPV infection (36.4%), suggesting a possible role in the carcinogenic process in the cells of the middle ear [88].

### 6.3. Anal and Vulvar Neoplasia

Recently, beta-HPV infection has been proposed as a possible contributor to the development of intraepithelial lesions in the vulva and anus. Sias et al. reported the presence of HPV9 in anal intraepithelial neoplasia I (AIN I), raising the question of whether beta-HPV genotypes contribute to the persistence of premalignant lesions in the anal epithelium or whether their presence is not a major risk factor [86]. On the other hand, Zulian et al. detected beta-HPV in anal samples of HIV-positive women, in which HPV5, 8, 38, and 111 were the most frequent genotypes. In addition, HPV118, 124, and 143 were found exclusively at anal sites, suggesting that immunocompromised individuals may be particularly susceptible to beta-HPV infection at mucosal anal sites; however, there was no significant association with premalignant lesions [81].

With respect to vulvar lesions, Lozar et al. reported beta-HPV infection in 26 p16-negative vulvar squamous intraepithelial lesion samples, and 42.3% of the samples were positive for beta-HPV, including HPV5, 9, 23, 24, 25, 36, 75, 105, 110, and 111 [84].

### 6.4. Colorectal Cancer

Emerging preliminary evidence has detected the presence of beta-HPV in colorectal cancer in Italy and the Czech Republic. Galati et al. detected beta-HPV DNA in 10% of adjacent nontumour tissues of Italian colorectal cancer patients, specifically HPV111 and 120. In the Czech population, beta-HPV genotypes, including HPV20, 24, 93, 98, 105, 124, 23, and 49, were detected in colorectal cancer by next-generation sequencing [85]. Whether these findings reflect active viral involvement in colorectal carcinogenesis or incidental colonization of mucosal tissue remains to be determined.

It is important to interpret these findings with caution, as they represent reported detection rather than a confirmed etiological role in carcinogenesis. Future mechanistic and prospective studies are necessary to determine the role of these viruses in mucosal cancers.

## 7. Addressing Beta-HPV Infection: Current Prevention and Prophylactic Approaches

### 7.1. Beta-HPV Screening

In cervical cancer, HPV genotyping is among the most important approaches to preventive screening. Currently, four primary HPV testing platforms have received FDA approval: Roche Cobas HPV, BD Onclarity HPV, Abbott Alinity m HR HPV, and Hologic APTIMA HPV. These tests are based on the detection of either HR-HPV DNA (cobas, Onclarity, Alinity) or mRNA of the *E6* and *E7* oncogenes (APTIMA), and recent regulatory advances have expanded their use to include self-collected vaginal specimens in clinical settings [94].

Currently available commercial beta-HPV detection tests are intended for research use only and remain considerably limited; only one test is available upon specific request: the RHA Kit Skin (beta) HPV from Cerba Research [95]. For research purposes, beta-HPV can also be detected using various technologies, including PCR combined with genotyping platforms and bead-based multiplex arrays, which are particularly useful for detecting beta-HPV in hair or skin swabs [81,96]. In situ hybridization (ISH) has been broadly used for tissue detection, especially to detect the transcriptionally active forms of the virus [85]. Moreover, quantitative PCR approaches have been used to determine the viral load in various EV lesion samples [97].

Recent advances in next-generation sequencing technologies, combined with the use of different sets of universal oligonucleotides reported in the literature, specifically PGMY and FAP, enable our research group to detect not only a wider range of alpha-HPV genotypes but also HPV genotypes from the beta and gamma genera in cervical cancer samples; this technology could enable broader genotype surveillance across diverse populations worldwide.

The presence of beta-HPV infections in oral, anal, vulvar, colorectal, and cervical cancer samples highlights the future possibility of developing new preventive screening tests that include beta-HPV detection, particularly among immunocompromised individuals, such as EV patients and organ transplant recipients. However, clinical evidence and data on the predictive value of beta-HPV infection in cancer remain limited, and this gap should be addressed before clinical beta-HPV screening can be implemented. Furthermore, most studies conducted to date have relied on tests covering a limited number of HPV types, predominantly those belonging to the alpha genus. Therefore, additional large-scale studies employing massive (next-generation) sequencing are needed worldwide to more comprehensively characterize beta-HPV infections and better define their role in carcinogenesis.

### 7.2. New HPV Vaccination Strategies

Current licensed HPV vaccines are based on L1 virus-like particles (VLPs) that restrict protection against specific alpha-HPV genotypes: Gardasil 9 (Merck, MSD) protects against HPV16, 18, 31, 33, 45, 52, and 58 (HR-HPV) and against HPV6 and HPV11 (LR-HPV); Cervarix (GSK) protects against the two most oncogenic genotypes, HPV16 and 18; and Gardasil 4 (Merck, MSD) protects against HPV6, 11, 16, and 18 [98].

Next-generation L2-based platforms (experimental vaccines with preclinical and clinical evidence) aim to broaden this coverage through cross-neutralizing antibodies against diverse HPV types. Among these, AAVLP (HPV16/31L2) is a platform consisting of virus-like particles derived from adeno-associated viruses that present HPV16 and HPV31 L2 epitopes. It has demonstrated cross-neutralizing antibodies against the alpha-HPV types HPV16, 18, 31, 45, 52, and 58 and in vivo protection against cutaneous and vaginal challenges with multiple alpha-HPV quasivirions; a phase I clinical study has been completed [98,99].

Although none of these vaccines offer protection against beta-HPV genotypes, anecdotal evidence suggests that the administration of some licensed vaccines may benefit cSCC patients. Two immunocompetent patients experienced reduced cSCC development following quadrivalent vaccine administration, and complete cSCC resolution was reported in another patient treated with combined systemic and intratumoral nonavalent vaccine administration [100]. Similar effects have been observed in premalignant lesions, including the resolution of epidermodysplasia verruciformis in an immunosuppressed renal transplant recipient following quadrivalent vaccine administration in conjunction with topical imiquimod and retinoids [101]. A possible explanation is that mucosal HR-HPV types are sometimes co-detected alongside beta-HPV genotypes in cSCC lesions of immunocompetent individuals; in such cases, antibodies generated by licensed vaccines against the alpha-HPV component may contribute to lesion clearance, even in the absence of direct beta-HPV coverage [102]. However, these findings remain anecdotal, and no prospective studies have confirmed the efficacy of licensed vaccines against beta-HPV-associated cSCC.

For this reason, new vaccines that offer protection against beta-HPV infections, particularly for high-risk individuals such as immunosuppressed individuals, have been proposed. These new vaccines should target the L2 minor capsid protein, which is less variable than L1 and harbors conserved N-terminal epitopes, specifically, the RG1 epitope (HPV16 L2 aa17–36) that elicits cross-neutralizing antibodies across diverse HPV genera, including beta-HPVs [102,103].

Among the platforms with specific preclinical evidence for beta-HPV coverage, RG1-VLP presents the RG1 epitope densely and repetitively on the surface of the HPV16 L1-VLP, eliciting cross-neutralizing antibodies against a broad range of HPV genotypes, including cutaneous beta-type HPV5, with in vivo protection against both mucosal and cutaneous HPV challenges [104]. Importantly, in a natural *Mastomys coucha* animal model, vaccination with RG1-VLPs cross-protected against infection with the naturally occurring cutaneous papillomavirus MnPV and significantly reduced skin tumor development in vivo, providing direct preclinical evidence for the potential of this platform in the prevention of beta-HPV-associated skin carcinogenesis [98,105].

In addition to prophylactic approaches, therapeutic vaccine platforms targeting the beta-HPV oncoproteins E6 and E7 are under investigation. These platforms, delivered as viral vectors, DNA/mRNA constructs, or nanoparticles, aim to elicit cytotoxic T-cell clearance of established lesions [98,106]. Current trials focus on cervical intraepithelial neoplasia and do not yet address its applicability in cSCC.

## 8. Conclusions

Beta-human papillomaviruses are ubiquitous skin commensals whose carcinogenic role differs fundamentally from that of alpha-HPV. While alpha-HPV drives cervical cancer through persistent viral integration and continuous oncoprotein expression, beta-HPVs possibly act as cofactors in a “hit-and-run” mechanism, transiently promoting the survival of UV-damaged keratinocytes through the activity of E6 and E7 and initiating oncogenic changes that persist independent of viral presence.

Notably, certain beta-3 genotypes, including HPV49, 75 and 76, display functional properties more commonly associated with alpha-HPVs, such as E6AP-dependent p53 degradation and keratinocyte immortalization, highlighting the biological diversity within the beta genus and underscoring the need for further investigation into the oncogenic potential of individual genotypes.

Additionally, beta-HPV contributes to carcinogenesis in cSCC alongside several interconnected factors, including genetic predisposition (as seen in EV patients), immune evasion, chronic inflammation, and environmental exposure (UV and tobacco). Given this context-dependent nature, the relevance of beta-HPV in oncogenesis appears to be concentrated in high-risk groups (namely, those with the aforementioned predisposing factors) rather than in the general population. Furthermore, current FDA-approved screening platforms are not designed to detect beta-HPV genotypes, and licensed vaccines provide no coverage against this genus, resulting in a significant gap in prevention for these vulnerable groups.

Finally, the presence of beta-HPV genotypes across multiple anatomical sites, including cervical, head and neck, anal, vulvar and colorectal carcinomas, has raised important questions about their possible etiological contribution beyond the skin. Nonetheless, the current evidence remains largely observational and methodologically heterogeneous, with most studies unable to establish causality or distinguish active infection from incidental colonization. It is important to mention that the beta-HPV types reported in at least three different cancer anatomical sites, in descending order of frequency, are HPV5, 38, 24, 8, 111, and 20. Clarifying whether beta-HPV plays a direct role in mucosal carcinogenesis or serves as a crucial cofactor for alpha-HPVs remains one of the most pressing knowledge gaps in the field.

## 9. Future Directions

Future research should prioritize large-scale epidemiological studies across diverse populations to establish whether beta-HPV plays a causal role in carcinogenesis and to identify which genotypes pose the greatest risk at specific anatomical sites. In parallel, the growing evidence linking beta-HPV to cSCC, and potentially to a broader spectrum of carcinomas, supports the case for integrating beta-HPV detection into preventive screening protocols, particularly for immunocompromised individuals such as organ transplant recipients and EV patients. Finally, the development of next-generation vaccines targeting beta-HPV, for which RG1-VLPs have already demonstrated promising cross-protective preclinical effects against cutaneous papillomavirus infection and tumor development, represents a critical frontier in skin cancer prevention.

## Figures and Tables

**Figure 2:**
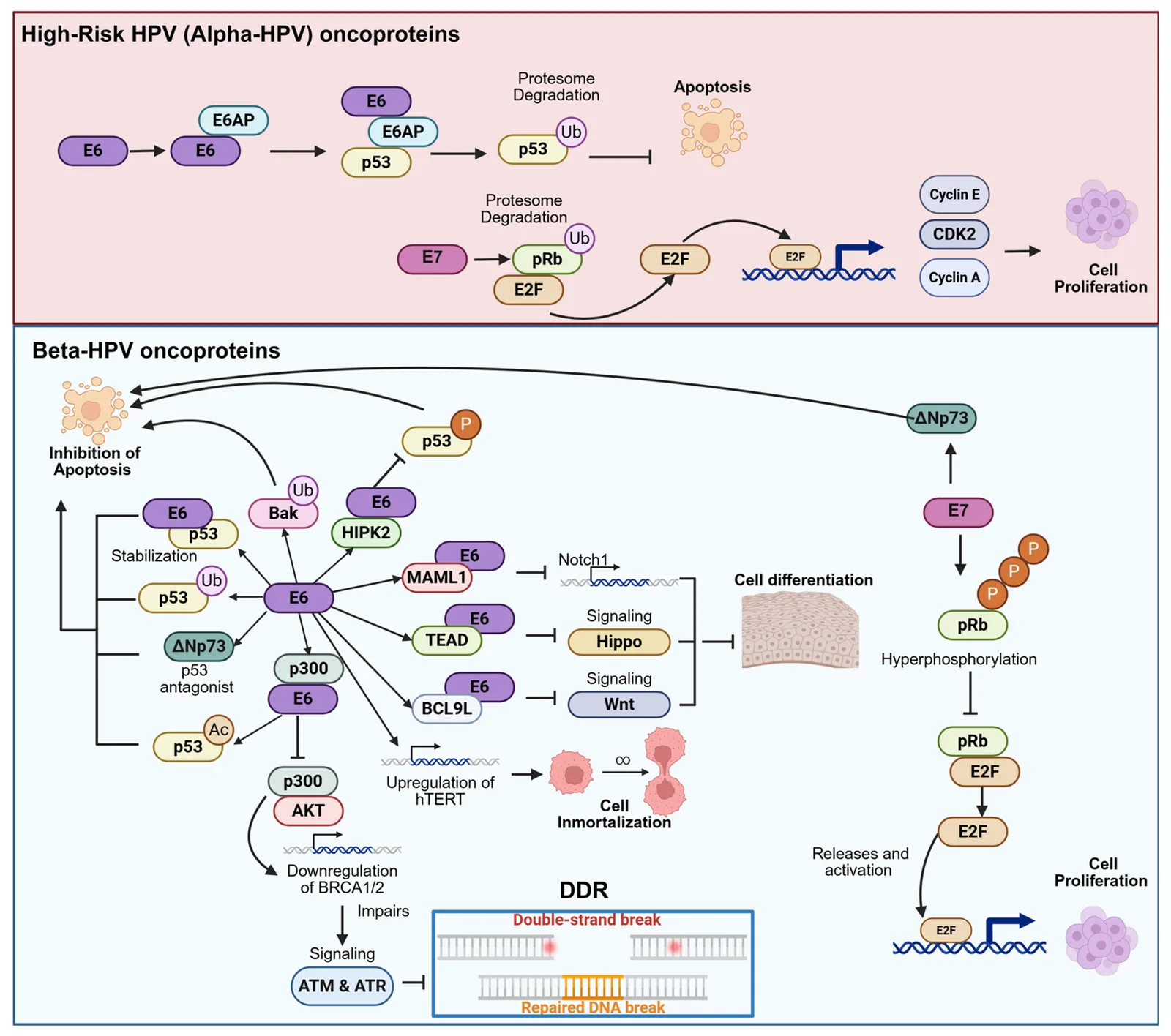
Oncogenic mechanisms of the alpha and beta-HPV oncoproteins E6 and E7. The red box represents the main oncogenic mechanism of alpha-HPV, while the blue box represents the oncogenic mechanisms of beta-HPV. Created in BioRender. Vizcarra Ramos, S. (2026) https://BioRender.com/fdnowut (accessed on 22 July 2026).

**Figure 3:**
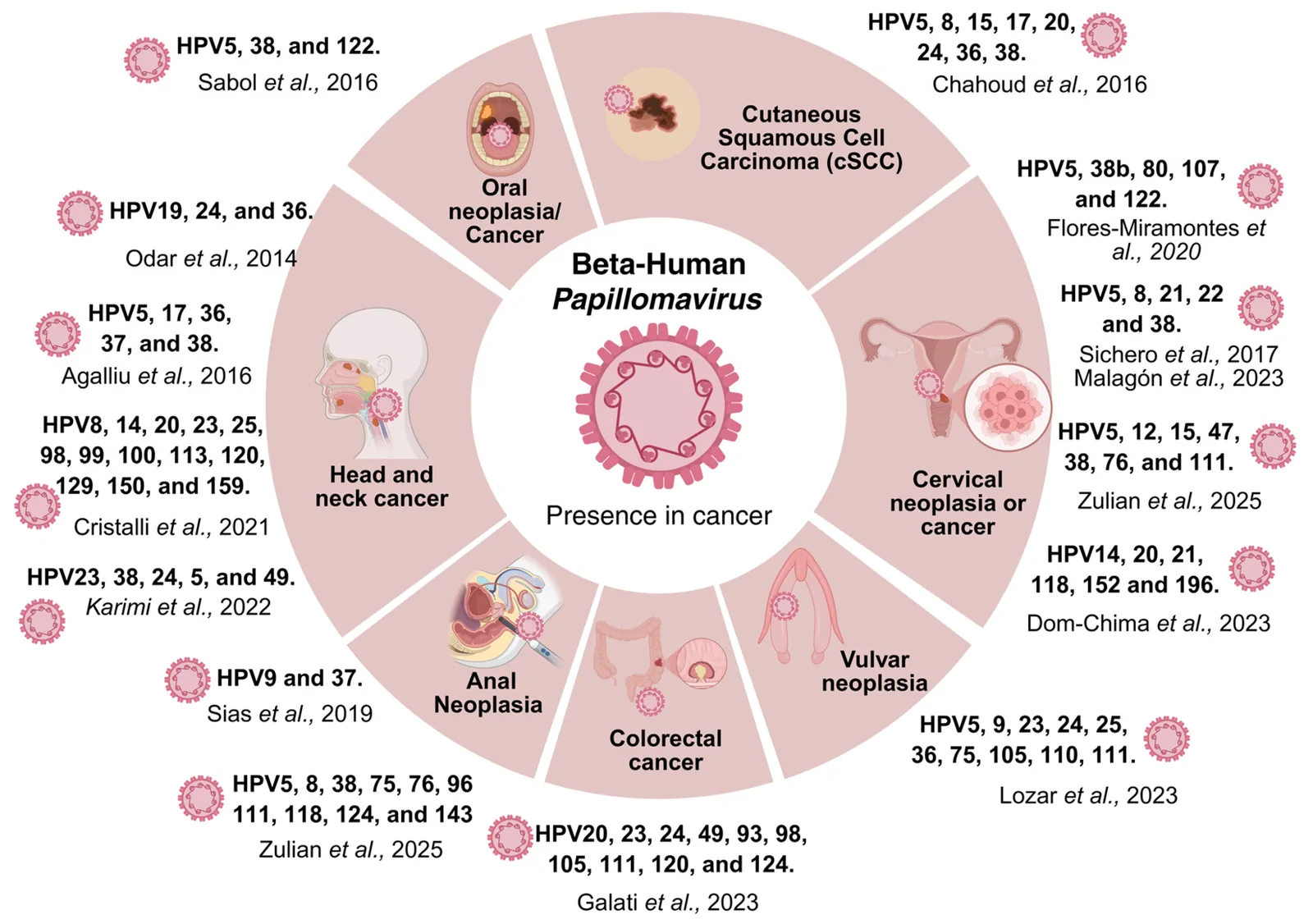
Detection of beta-human papillomavirus in cancer. The detection of beta-HPV infection across different anatomical sites in individuals with different types of cancer was performed according to the methods in the referenced publications [9,79,80,81,82,83,84,85,86,87,88,89,90,91]. Created in BioRender. Vizcarra Ramos, S. (2026) https://BioRender.com/mdy8hc7 (accessed on 22 July 2026).

**Table 1:** Mechanisms mediated by E6/E7 of beta-HPV.

HPV Genotype(s)	Species	Protein	Mechanism(s)	Study Model	Reference
5, 8	beta-1	E6	p300/AKT destabilization	Primary HFK ^1^	[48]
5, 8	beta-1	E6	ATR inhibition	Primary HFK	[49]
5, 8	beta-1	E6	BRCA1/2 downregulation	Primary HFK	[39]
5, 8, 38	beta-1/2	E6	Genomic instability	Primary HFK	[40]
5, 8, 20, 22, 38, 76, 92, 96	beta-1/2/3/4/5	E6	Bak degradation	Primary HFK	[42]
8, 17, 38, 76, 92	beta-1/2/3/4	E6	Notch repression (MAML1)	Primary HFK	[43,44]
8	beta-1	E6	Hippo/Wnt dysregulation	n/Tert-1 ^2^	[45]
17, 38, 92	beta-2/4	E6	p53 stabilization	n/Tert-1	[35]
23, 38	beta-2	E6	HIPK2 binding	H1299 ^3^	[34]
38	beta-2	E6	p300 binding, p53 acetylation blockade	Primary HFK	[41]
38	beta-2	E6	ΔNp73 accumulation	Primary HFK; transgenic mice	[36]
38, 49, 75, 76	beta-2/3	E6/E7	hTERT-mediated immortalization	Primary HFK	[46]
49, 75, 76	beta-3	E6	E6AP-mediated p53 degradation	Primary HFK	[50]
38, 49, 75, 76	beta-2/3	E7	pRb hyperphosphorylation	Primary HFK	[37,46]
38	beta-2	E7	ΔNp73 accumulation; IKKβ/ΔNp73α/DNMT1/EZH2 complex (PIG3 silencing)	HPK38 ^4^	[51]

^1^ HFK, primary human foreskin keratinocyte. ^2^ n/Tert-1, hTERT-immortalized (nontumorigenic) human keratinocyte line. ^3^ H1299, p53-null human lung carcinoma cell line. ^4^ HPK38 and HFK cells immortalized by stable expression of HPV38 E6/E7.

**Table 2:** Beta-HPV mechanisms in collaboration with environmental and immune-related host factors.

HPV Genotype(s)	Host/ Environmental-Factors	Mechanism(s)	Study Model	Reference
5, 8	*EVER1/EVER2* mutations	Loss of epithelial antiviral response	EV patients	[7]
5, 8	EVER1/EVER2 disfunction	Impaired zinc balance in keratinocytes/lymphocytes	Human keratinocytes; T/B lymphocites	[60]
5	EVER2 dysfunction	LCR blockade loss (JNK-dependent)	EVER2- deficient keratinocytes	[61]
5, 8	EVER2 dysfunction	TRAIL/TNF-α-dependent apoptosis of infected cells	Human Keratinocytes	[61]
beta-HPV (genus-wide)	*CIB1* mutations	Loss of keratinocyte-intrinsic restriction	EV patients	[62]
9, 15, 17	*SNP* rs9264942-T	Altered HLA-C/CTL activation	Humans; beta-HPV seropositivity cohort	[63]
38	*E6/E7* (ΔNp73α)	TLR9 transcriptional repression	HPV38 E6/E7 keratinocytes (HFK)	[64]
38	UV radiation	Loss of p53/c-Jun-mediated TLR9 activation	UV-irradiated HPV38 E6/E7 HFK	[65]
38	*E6/E7* (ΔNp73α/PKR/DNMT1)	IL-18 transcriptional silencing	HPV38 E6/E7 keratinocytes (“38HK”) + UVB	[66]
38	*E6/E7* (*STAT1* downregulation)	MHC downregulation (*IRF1/TAP*)	HPV 38 E6/E7 HaCaT	[67]
5, 8	E6 (CBP-IBiD binding)	IRF3/IFN-β inhibition	E6-transfected cell lines	
8	E6	cGAS-STING attenuation	HPV8 E6 hTERT-HFK	[68]
8	E7 (C/EBPβ)	*CCL20* suppression, Langerhans cell migration loss	NHK; EV lesional skin	[69]
5	Antiviral cytokine response	*IL-6, IL-8, ICAM-1, CCL2* upregulation	Primary keratinocytes; HaCaT	[70]
8	CCL2/monocyte recruitment	CCR2-dependent infiltration, causal in cSCC	K14-HPV8-E6; Ccr2^−/−^ mice	[71]
8	Calprotectin (*S100A8/A9*)	Neutrophil recruitment	n/Tert-1	[72]
49	Tobacco (4NQO)	Upper digestive tract carcinogenesis susceptibility	K14 HPV49 E6/E7 Tg mice	[73,74]

## Data Availability

No new data were created or analyzed in this study. Data sharing is not applicable to this article.

## Abbreviations

The following abbreviations are used in this manuscript:

HPV	Human Papillomavirus
NMSC	Nonmelanoma Skin Cancer
cSCC	Cutaneous Squamous Cell Carcinoma
AIN	Anal Intraepithelial Neoplasia
MESCC	Middle Ear Squamous Cell Carcinoma
HNSCC	Head and Neck Squamous Cell Carcinoma
pRb	Retinoblastoma protein
MnPV	Mastomys natalensis Papillomavirus
